# Evaluation of lung homogeneity in neonates and small infants during general anaesthesia using electrical impedance tomography: a prospective observational study

**DOI:** 10.1016/j.bjao.2024.100344

**Published:** 2024-09-21

**Authors:** Vanessa Marchesini, Sebastian Corlette, Suzette Sheppard, Andrew Davidson, David Tingay

**Affiliations:** 1 Department of Anaesthesia and Pain Management, The Royal Children's Hospital, Parkville, Melbourne, VIC, Australia; 2 Department of Anaesthesia, Murdoch Children's Research Institute, Parkville, Melbourne, VIC, Australia; 3 Department of Paediatrics, University of Melbourne, Melbourne, VIC, Australia; 4 Department of Neonatology, Royal Children's Hospital, Parkville, Melbourne, VIC, Australia; 5 Neonatal Research, Murdoch Children's Research Institute, Parkville, VIC, Australia

**Keywords:** electrical impedance tomography (EIT), general anaesthesia, infant, mechanical ventilation, neonate, regional ventilation

## Abstract

**Background:**

Prolonged mechanical ventilation can create heterogeneous ventilation patterns, which increase the risk of lung injury in infants. However, little is understood about the risk of brief exposure to mechanical ventilation during anaesthesia. The aim of this prospective observational study was to describe the regional pattern of lung ventilation during general anaesthesia in healthy neonates and infants, using electrical impedance tomography.

**Methods:**

Twenty infants (age 3 days to 12 months), without known lung disease and receiving general anaesthesia with endotracheal intubation for supine positioned surgery, were included in the study. Anaesthesia and ventilation management was at the discretion of the treating clinician. Standardised lung imaging using electrical impedance tomography was made at six time points during anaesthesia from induction to post-extubation. At each time point, the gravity-dependent and right–left lung centre of ventilation was calculated.

**Results:**

Tidal ventilation favoured the dorsal lung regions at induction, with a median (inter-quartile range) centre of ventilation (CoV) of 58.2 (53.9–59.3)%. After intubation, there was a redistribution of ventilation to the ventral lung, with the greatest change occurring early in surgery: CoV of 53.8 (52.3–55.2)%. After extubation, CoV returned to pre-intubation values: 56.5 (54.7–58)%. Across all time points, the pattern of ventilation favoured the right lung.

**Conclusions:**

General anaesthesia creates heterogenous patterns of ventilation similar to those reported during prolonged mechanical ventilation. This potentially poses a risk for lung injury that may not be recognised clinically. These results suggest the need to better understand the impact of general anaesthesia on the developing lung.

**Clinical trial registration:**

Australian New Zealand Clinical Trials Registry (ACTRN 12616000818437, 22 June 2016).

Mechanical ventilation is often required to support impaired respiratory function, but when inappropriately applied can cause ventilator-induced lung injury (VILI).^1^^,^^2^ One of the principal factors related to VILI is uneven delivered ventilation. Uneven ventilation allows the potential for multiple mechanisms of injury to occur concurrently, atelectasis, and shear-force injury in poorly ventilated regions and volutrauma in neighbouring overly ventilated regions. This risk is well understood in the ICU where prolonged periods of mechanical ventilation are often required, but little is known about the impact of short exposure of mechanical ventilation during anaesthesia. Some populations are at greater risk, particularly infants owing to their undeveloped lungs.^3^ In preclinical studies, even brief periods of inappropriate mechanical ventilation create lung injury and initiate the cascade to later pro-inflammatory states in the preterm lung.^4^, ^5^, ^6^ The clinical implications of such exposure are still unknown. The interaction between lungs and general anaesthesia in the paediatric population has mainly been evaluated using global pulmonary mechanics and radiological imaging, and shown a decreased ventilation distribution and high incidence of atelectasis.^7^, ^8^, ^9^, ^10^, ^11^ Common bedside tools such as peripheral oxygen saturation (SpO_2_), end-tidal CO_2_, and dynamic lung compliance lack the sophistication to determine whether and where uneven ventilation may be present. In the past decade, electrical impedance tomography (EIT) that measures the impedance of the thorax through a belt placed around the patient's chest has been developed.^12^^,^^13^ Being a noninvasive, radiation-free monitoring tool that allows real-time continuous imaging of ventilation at the bedside, EIT can be used to easily assess lung homogeneity in different clinical settings and populations.

The aim of this prospective observational cohort study was to describe the regional (gravity-dependent and right–left) lung ventilation during different stages of general anaesthesia in neonates and infants with healthy lungs pre-anaesthesia.

## Methods

The study was conducted at the Department of Anaesthesia and Pain Management, The Royal Children's Hospital, Melbourne, Australia. The Human Research Ethics Committee at The Royal Children's Hospital approved the study (HREC 36008A, 28 January 2016). The recruitment process was conducted between March 2016 and May 2018. Prospective written informed consent was obtained from patients' parents. Neonates and infants (<1 yr of age) who underwent general anaesthesia requiring endotracheal intubation with a cuffed tube and positioned supine during surgery were considered eligible for the study. Patients with known lung disease, who had previously received mechanical ventilation or with a history of prematurity (<32 weeks completed gestational age at birth) were excluded. Patients undergoing cardiac or thoracic surgery were also excluded as the placement of the EIT belt would interfere with the surgical field.

Anaesthesia and ventilation management was not standardised and at the discretion of the treating anaesthetist. After induction of anaesthesia, lung ventilation and oxygenation was supported using a facemask and hand-bag technique via a T-piece circuit. After tracheal intubation, using a cuffed tracheal tube appropriate to the size of the patient, ventilation was initially supported manually using a T-piece circuit. During surgery, pressure settings and mechanical ventilation modalities were at the discretion of the anaesthetist. On completion of surgery, anaesthesia was ceased, and the patient's trachea extubated after the return of spontaneous breathing. Ventilation and oxygenation were supported using a facemask and T-piece circuit after extubation if required.

### EIT measurements

After induction and before intubation, a correctly sized non-adhesive infant EIT electrode belt (Sentec AG, Landquart, Switzerland) was placed around the chest of the patient at the level of their nipples using our previously described approach.^13^ The EIT belt was coated with ultrasound gel before application to improve electrical conductivity. The EIT belt was connected to the EIT system (BB2 or Pioneer systems; Sentec AG) and signal and imaging quality of the EIT electrode belt determined. If signal quality was inadequate, the belt position was altered, additional ultrasound gel applied, or the belt replaced as appropriate.

Once suitable EIT signal quality was confirmed, 2-min EIT recordings (sampling rate of 48 frames per second) were obtained at six different time points during periods of stable clinical care and no patient handling to represent different time points during general anaesthesia: T0, spontaneous breathing with face mask after induction of anaesthesia; T1, hand-bag ventilation via a T-piece circuit after intubation; T2, mechanical ventilation at the start of surgery (defined as within 5 min of commencing surgery); T3: mechanical ventilation during surgery (defined as within 20–30 min from the start of surgery depending on surgical interventions to allow stable ventilation); T4: mechanical ventilation at the end of the surgical procedure (defined as not more than 5 min after the end of surgery); T5, spontaneous breathing after extubation.

If the duration of surgery was expected to be less than 30 min, EIT measurements between 10 and 15 min from the start of surgery were made and used as EIT data for time point T3. The measurement at T0 was considered the baseline ventilation pattern for that infant.

### Data acquisition and analysis

Details of the patients' medical history were collected along with their age, sex, weight, gestational age at birth, comorbidities, type and duration of surgery, episodes of desaturation or any critical events during anaesthesia. Respiratory support values during surgery (including pressure and volume settings, type and mode of mechanical ventilation) were also collected.

Raw EIT data were reconstructed in a custom-built infant imaging package (IBEX-neo; Sentec AG) using a finite element model of the human infant chest according to consensus guidelines.^13^^,^^14^ This model automatically excludes non-lung containing chest regions from analysis.^15^ The final EIT tidal data were then manually assessed for movement artifact or poor signal quality. The first 30 s of continuous artifact-free tidal ventilation from each stage of general anaesthesia was chosen for analysis. If no such period was available, a shorter period of artifact-free data was included with at least 15 consecutive inflations. If there were fewer than 15 inflations, the data for that stage were excluded. Data were not included in the analysis unless the above criteria were met in three or more stages for the same patient.

**Fig 1:**
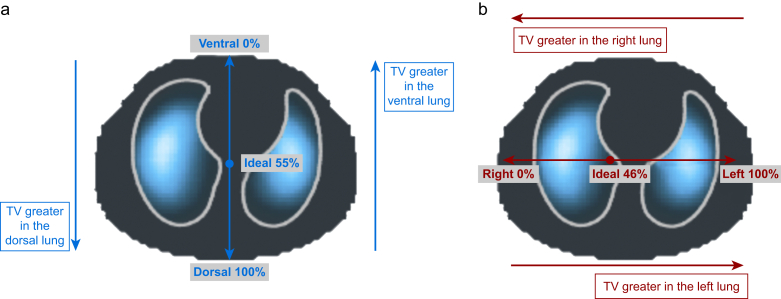
Representative functional electrical impedance tomography (EIT) image of homogenous ventilation along the ventral–dorsal (a) and right–left plane (b) with centre of ventilation (CoV) for each plane shown. EIT creates a cross-sectional slice image of ventilation within the field of imaging (electrode belt). Magnitude of tidal volume is represented using a colour scale from white (most ventilation) to grey (least ventilation). The ideal CoV occurs at 55% of the distance between the most ventral (0%) and dorsal (100%) lung, and 46% of the distance between the most right (0%) and left (100%) lung regions. TV, tidal ventilation.

The geometric centre of ventilation (CoV) was calculated for each final EIT measurement. The coordinates of the CoV are defined along the gravity-dependent ‘ventral-to-dorsal’ axis (CoV_VD_) and along the horizontal ‘right-to-left’ axis (CoV_RL_). The CoV is a single value describing the geometric mean of tidal volume distribution along a single plane (dorsal/ventral or right/left). It is represented as a percentage to define the geographic distance along the plane of imaging. The lung regions furthest to the right and most ventral are defined as 0%, and the lung regions furthest to the left and most dorsal are defined as 100%. Thus, if tidal ventilation is greater in the right lung than in the left, the CoV_RL_ will be <50% and the magnitude informs as to the weighting of heterogeneity (Fig. 1). To account for the uneven sizes of lung mass in each region (more tissue for ventilation in the central regions and right lung), the CoV is referenced to an ‘ideal’ CoV which would represent perfectly homogeneous ventilation along the chest plane of interest. This is 46% for CoV_RL_ and 55% for CoV_VD_ in the infant model used in this study.^16^^,^^17^ Variation of 2% or more might be of clinical significance.^13^

### Statistical analysis

At the time of study design, patterns of regional ventilation were not previously reported in infants undergoing surgery under general anaesthesia. Thus, a formal sample size calculation was not possible. About 20–25 infants have been shown to identify different patterns of regional tidal ventilation using EIT in NICU settings.^18^, ^19^, ^20^ A feasibility sample size of 30 patients was chosen to allow for incomplete data or technical issues.

Primary analysis was limited to simple descriptive statistics. The study design did not warrant analysis for associations or hypothesis testing. After completion of data collection, because of variability in ventilation modalities, further descriptive analysis was performed to explore regional ventilation pattern in two separate groups. Data were separated between pressure-controlled ventilation (PCV) and spontaneous ventilation modes. Spontaneous ventilation modes included both pressure support ventilation (PSV) and spontaneous breathing. The aim of this grouping was to explore the impact of different levels of diaphragmatic activation on lung ventilation. The sample size did not allow for further subgroup analysis to investigate the impact of different ventilation and anaesthesia strategies or type of surgery. The data are described as absolute and relative frequencies for the categorical variables and as median and inter-quartile range for continuous variables. The analyses were performed with the R statistical software (R Foundation for Statistical Computing, Vienna, Austria).

## Results

Thirty patients were recruited for this study with data from 20 available for analysis. Six patients were excluded at the time of recruitment because of device failure or modification of the surgical plans not meeting the inclusion criteria. Four patients were excluded from analysis because of insufficient data. Patient characteristics and a summary of respiratory support are presented in Table 1. Of note, positive end-expiratory pressure (PEEP) was used in 16 out of 18 patients who received mechanical ventilation and no recruitment manoeuvres were documented. Inhalational induction with sevoflurane was used for 18 of the 20 patients. The remaining two patients received i.v. induction with propofol. Neuromuscular block was used in seven patients before intubation. There were no episodes of desaturation, severe bradycardia, or critical events during the study period.

**Table 1:** Patient characteristics. Data are presented as *n* (%) or median (inter-quartile range). ENT, ear nose, and throat; PEEP, positive end-expiratory pressure.

Patient characteristics	*N*=20
Female, *n* (%)	12 (60)
Age at surgery (weeks)	32.4 (22.3–45.9)
Weight at surgery (kg)	7.55 (5.95–8.95)
Gestational age at birth (completed weeks)	40 (37.5–40)
Duration of surgery (min)	44 (23.3–164)
**Anaesthesia details**	
Type of surgery, *n* (%)	
Abdominal	8 (40)
Urology	3 (15)
Orthopaedic	1 (5)
Maxillofacial	6 (30)
ENT and ophthalmology	2 (10)
ASA physical status, *n* (%)	
1	13 (65)
2	5 (25)
3	2 (10)
**Respiratory support**	
Mode of ventilation, *n* (%)	
Pressure-controlled ventilation	12 (60)
Pressure support ventilation	6 (30)
Spontaneous breathing	2 (10)
PEEP (cm H_2_O)	4.5 (4–5)
Inspiratory pressure (cm H_2_O)	15.5 (10–17.5)
Tidal volume (ml kg^−1^)	7.9 (6–9.8)

### Lung centre of ventilation

**Fig 2:**
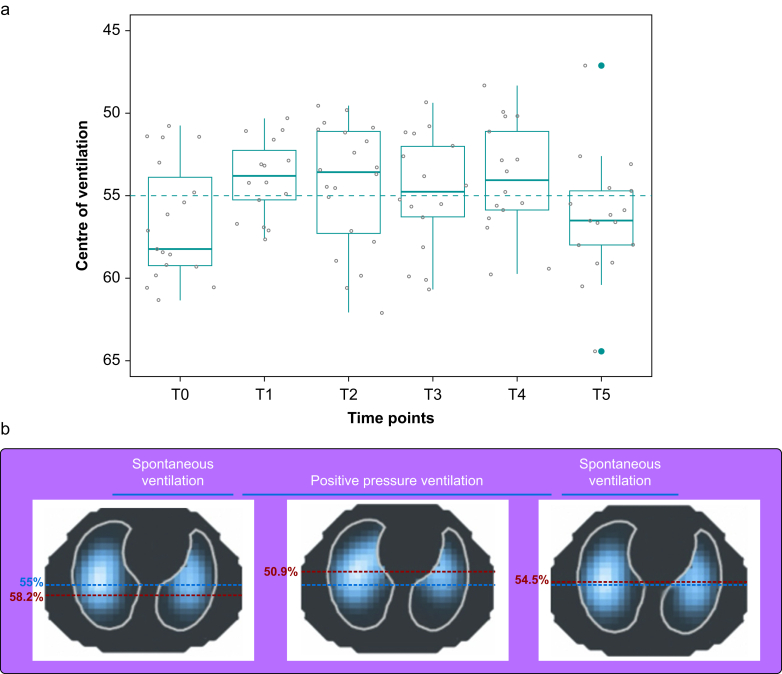
(a) Centre of ventilation along the ventral–dorsal plane (CoV_VD_) at six anaesthesia time points: T0, spontaneous breathing with face mask at induction of anaesthesia; T1, hand-bag ventilation via T-piece after intubation; T2, mechanical ventilation at the start of surgery; T3, mechanical ventilation during surgery; T4, mechanical ventilation at the end of surgery; T5, spontaneous breathing after extubation. Boxes represent median and inter-quartile range (IQR), lines represent maximum and minimum values, and bold green dots are individual points representing outliers. Grey dots represent individual patient values. Dashed line represents the ideal CoV_VD_ (55%; homogenous gravity-dependent ventilation). Tidal ventilation favoured the more gravity-dependent (dorsal) lung regions during induction (T0), with a median (IQR) CoV_VD_ of 58.2 (53.9–59.3)%. After intubation, there was a redistribution of ventilation to the non-dependent (ventral) lung, with the greatest change occurring at the start and during surgery (T1 and T2): 53.8 (52.3–55.2)% and 53.6 (51.1–57.3)%, respectively. After extubation (T5), CoV_VD_ returned to pre-intubation values: 56.5 (54.7–58.0)%. (b) Representative functional electrical impedance tomography (fEIT) images from patient 26. Magnitude of tidal volume is represented using a heatmap colour scale from white (most ventilation) to grey (least ventilation). Blue dashed line illustrates the ventral–dorsal plane of homogeneous ventilation (CoV_VD_ 55%). Red dashed line illustrates the measured ventral–dorsal centre of ventilation (CoV_VD_) for patient 26. The fEIT images show a loss of dorsal ventilation during positive pressure ventilation from spontaneous breathing once intubated and receiving positive pressure ventilation. Greater dorsal lung ventilation returned after extubation.

**Fig 3:**
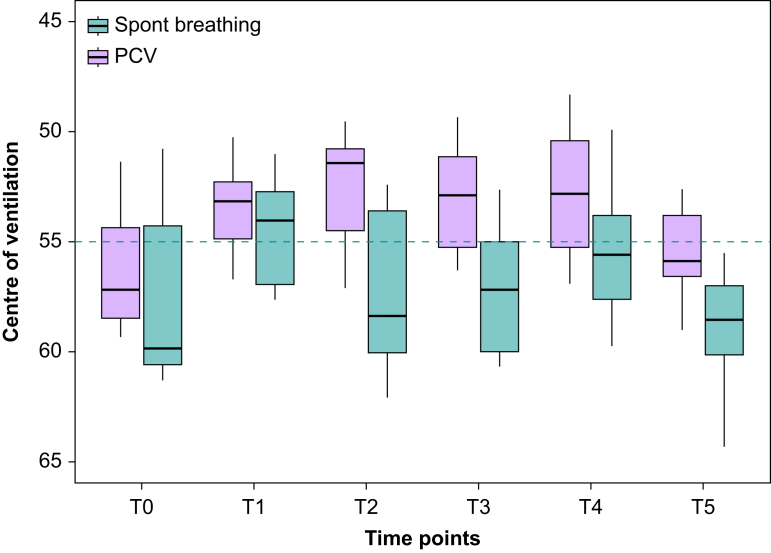
Centre of ventilation along the ventral–dorsal plane (CoV_VD_) during apnoeic respiratory support with pressure-controlled ventilation (‘PCV’; *n*=12, pink bars) compared with pressure support ventilation and manual spontaneous ventilation (‘Spont breathing’; *n*=8, green bars) at six anaesthesia time points (see Fig. 2 for description). The shift of lung ventilation towards ventral regions during surgery (T1 to T4) is more pronounced in the PCV group than in the spontaneous (Spont) breathing group where the pattern of ventilation remains mainly in the dorsal regions. In particular, at T2 CoV_VD_ in the ‘Spont breathing’ mode was 58.4 (53.6–60.0)% compared with 51.4 (50.8–54.5)% in the ‘PCV’ mode. Similarly, at T3 CoV_VD_ in the ‘Spont breathing’ mode was 58.1 (55.0–60.0)% compared with 52.9 (51.2–55.2)% in the ‘PCV’ mode.

**Fig 4:**
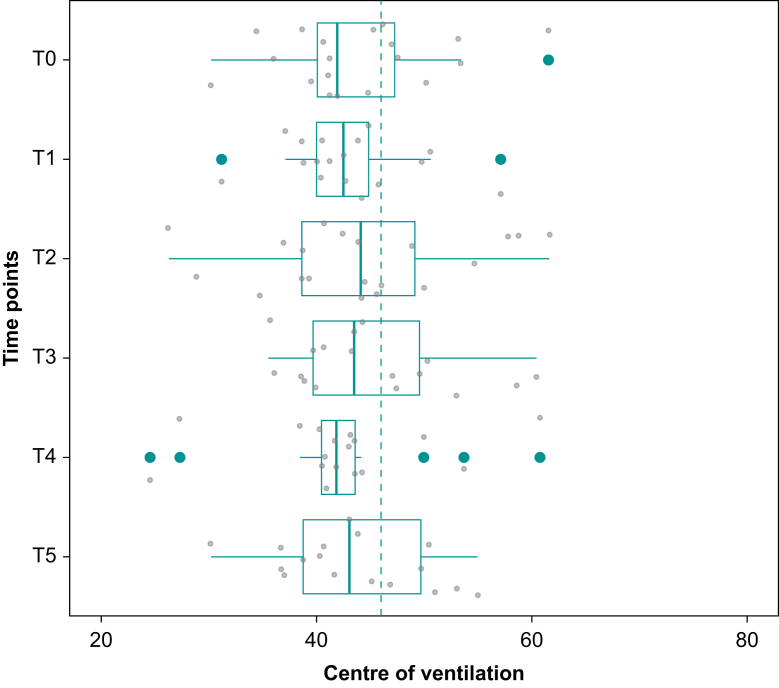
Centre of ventilation along the right–left plane (CoV_RL_) at six anaesthesia time points: T0, spontaneous breathing with face mask at induction of anaesthesia; T1, hand-bag ventilation via T-piece after intubation; T2, mechanical ventilation at the start of surgery; T3, mechanical ventilation during surgery; T4, mechanical ventilation at the end of surgery; T5, spontaneous breathing after extubation. Boxes represent median and inter-quartile range, lines represent maximum and minimum values, and bold green individual points represent outliers. Small grey circles represent individual patient values. Dashed line represents the ideal CoV_RL_ (46%). The right-to-left centre of ventilation is shifted towards the right lung with minimal variation in all time points (T0 41.9 [39.5–47.6]%; T1 42.5 [40.0–44.8]%; T2 44.1 [38.6–49.4]%; T3 43.5 [39.7–49.6]%; T4 41.8 [40.4–43.6]%, T5 43.0 [38.7–49.7]%).

**Fig 5:**
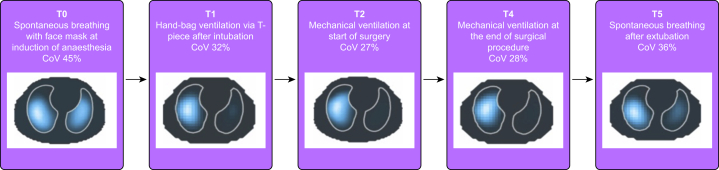
Representative functional electrical impedance tomography (fEIT) images from patient 7 with resultant right–left centre of ventilation (CoV_RL_) values to demonstrate potential right bronchus intubation. At T0, when patient was spontaneously breathing with face mask support, the right–left plane ventilation is homogeneous. At T1, after intubation, there is almost complete loss of ventilation on the left lung that persists at T2 and T4. After extubation (T5), there is a partial return of ventilation in the left lung, but not yet at T0 pattern. No signs of clinical deterioration were recorded during the anaesthesia. EIT recording at T3 was excluded during *post hoc* analysis because of poor data quality.

Ventilation distribution along the ventral–dorsal and right–left plane was not uniform and highly variable (Supplementary Figs S1 and S2). Tidal ventilation favoured the more gravity-dependent (dorsal) lung regions during induction, shifted towards ventral regions during surgery, and returned to pre-intubation values after extubation (Fig. 2 describes the pattern of CoV_VD_ at each time point). This pattern of lung ventilation towards the non-dependent region was accentuated during mandatory ventilation (PCV) compared with spontaneous ventilation modes (PSV and spontaneous breathing; Fig. 3). Ventilation distribution along the right–left plane showed an overall shift towards the right lung at all time points (Fig. 4). Three out of 20 patients were noted to have a change in CoV_RL_ of more than 10% from the T0 (pre-intubation) value to after intubation (Fig. 5; Fig. 1 online video). Such a large change is unlikely to be physiological and suggests unrecognised single-bronchus intubation. In all three patients, CoV_RL_ returned to T0 values after extubation and none experienced abnormal vital signs or clinical features suggestive of one-lung ventilation.

## Discussion

In this prospective observational study, we describe the pattern of ventilation during different clinical phases of general anaesthesia in neonates and infants with no known lung disease. In our study population, mechanical ventilation during anaesthesia resulted in a redistribution of ventilation towards the non-gravity-dependent lung regions when compared with spontaneous breathing without atracheal tube before intubation and after extubation.

We studied infants <1 yr old. Similar patterns of ventilation redistribution have been reported in studies of anaesthetised children ranging from 9 weeks to 10 yr.^21^^,^^22^ In these studies, mechanical ventilation during anaesthesia resulted in redistribution of ventilation from dorsal to ventral regions, with restoration of dorsal-dominant ventilation after extubation. A change in ventilation distribution towards the dependent lung after induction of anaesthesia was also reported in infants and children undergoing elective cardiac surgery.^23^ These findings are not surprising but likely underappreciated in the healthy lung without impairment of oxygenation. General anaesthesia promotes gravity-dependent atelectasis formation through several mechanisms: decrease in ventilation trigger from loss of central respiratory control, inactivation of intercostal and diaphragmatic muscle activity, and resorption of alveolar gas during inhalation of a high fraction of inspired oxygen.^24^ In addition, neonates and small infants have specific physiological characteristics that increase the risk of overdistention in the ventral lung region and atelectasis in the dorsal region. They have a reduced pulmonary elastic recoil, a closing airway pressure near or below functional residual capacity, and higher chest wall and lower lung compliance.^3^

The different pattern of ventilation when spontaneous breathing was present (maintained towards the dorsal region) compared with the controlled mechanical ventilation (CoV shifted towards the ventral region) is also understandable.^19^^,^^25^ Despite the lack of standardised use of neuromuscular block in our patients, it is likely that the common anaesthetic agents (such as propofol, opioids, and sevoflurane) might have considerably reduced the degree of diaphragmatic activation until the abolishment of the respiratory muscle activity. The diaphragm plays an important role in dorsal lung ventilation.^18^ There is more diaphragm in the dorsal part of the chest and its contraction is most effective in the dorsal region of the chest where the posterior muscle fibres shorten the most. This helps to counterbalance the gravity impact of overlying lung mass on dorsal lung opening pressures.^26^ The role of spontaneous diaphragmatic activity was shown to be an important aspect of gravity-dependent ventilation homogeneity in relatively stable neonates in the ICU.^18^ It is in part on this basis that supporting spontaneous diaphragmatic activity during positive pressure ventilation is rec ommended for lung protection in neonates in the NICU.^27^^,^^28^ In the anaesthetic setting, the use of sedative drugs or anaesthetic drugs may differentially alter respiratory muscle function compared with the NICU setting, and therefore lead to a variable degree of lung heterogeneity even during breathing.^29^

Our study also showed that the right lung was preferentially ventilated irrespective of the anaesthesia time point, with little variation between spontaneous and mechanical ventilation. Similar findings have been reported using EIT in observational studies in the delivery room and NICU during invasive respiratory support, noninvasive ventilation, and spontaneous breathing.^19^^,^^20^^,^^30^ As the ‘ideal’ CoV_RL_ measure already accounts for the discrepancies in lung size between the right and left lung, this finding is likely to result from a dynamic interaction between the gas flow and upper airways. The gas flow might preferentially follow the pathway of less resistance through the wider but shorter and more vertical right main bronchus compared with the left bronchus, especially during the higher-flow states of mechanical ventilation.^31^ This observation is interesting, but its clinical relevance remains unclear.

Single-lung ventilation was incidentally found in three infants in whom it was not reported clinically. The possible causes for this finding include inadvertent mainstem bronchus intubation, mucus plugging, anatomical abnormalities, and severe atelectasis or pneumothorax. Bedside assessment of tracheal tube position (such as auscultation, chest wall movement, capnography) is often unreliable in small children, and small changes of neck and head position might affect ventilation, especially when the tracheal tube is very close to the carina.^32^, ^33^, ^34^ In conjunction with lung ultrasound,^35^ EIT may be a feasible, easy, and noninvasive tool to verify that a tracheal tube is positioned correctly and exclude any other causes of one-lung ventilation, especially in neonates where airway management is difficult.^36^ This combined approach can be highly valuable as it might help detect abnormal lung physiology and guide ventilation strategy.

Overall, the ventilation distribution exhibited high intrasubject and intersubject variability. This might be owing to a lack of standardisation of anaesthesia care and ventilation strategies, but it may also reflect the physiologically expected innate variability in breath-to-breath respiratory function. Different types of surgery, the variable use of neuromuscular block, inspired fraction of oxygen, PEEP, and ventilation settings within our cohort might have played a role in the ventral-to-dorsal and right-to-left ventilation distribution variability and require investigation in a standardised manner.

More important than variability was the observation that gravity-dependent lung heterogeneity can occur despite no major oxygenation or ventilation complications. This suggests that commonly used respiratory monitoring is unlikely to provide detailed information on effectiveness and safety of delivered respiratory support. Unevenness of ventilation is a recognised factor in VILI in the ICU setting. However, it remains unknown if brief exposure to lung heterogeneity during general anaesthesia is injurious to a healthy infant lung and if it is associated with clinically meaningful adverse outcomes. As currently there is no readily available method for assessing lung homogeneity in daily anaesthesia practice, it is possible that the potential injury of mechanical ventilation is unrecognised. At the very least, EIT offers the potential to better inform clinicians of injurious ventilation patterns in patients under their care and enable them to optimise the respiratory strategy (such as by altering PEEP) and monitor the impact of those changes.

There are some study limitations. This study was observational and included a small number of patients. The high incidence of patient exclusions was from the nature of the EIT device at the time of recruitment, when the EIT system was customised by the manufacturer only for research use in infants and neonates. Ideally a preoperative baseline reference image before the induction of anaesthesia would have been useful. The comparison between baseline ventilation, spontaneous breathing under anaesthesia, and controlled ventilation might exhibit a larger difference, given the variable degree of respiratory muscle involvement. Clinically relevant limitations of the study can be identified in the lack of standardised ventilation technique, different type of surgical procedures, and the absence of more refined data on gas exchange and intrapulmonary shunt.

Our study described the pattern of ventilation during general anaesthesia in neonates and infants. At this stage, no definitive conclusions regarding presence and degree of lung injury or long-term respiratory outcomes can be made. Further studies should focus on the impact of different ventilation strategies and the use of neuromuscular blocking agents on lung homogeneity, and include data on gas exchange to better delineate the clinical effect of lung heterogeneity.

## Authors’ contributions

Study design/planning: VM, DT, SS, AD.

Patient recruitment: VM, SC.

Data analysis and interpretation: VM, DT.

Writing up the first draft of the paper: VM, DT.

Revising the manuscript critically for important intellectual content: all authors.

Final approval of the version to be published: all authors.

## Funding

Support was provided solely from institutional and departmental sources.

## Declaration of interest

The authors declare they have no conflict of interest.

## Appendix A Supplementary data

Multimedia component 1 Multimedia component 1

Video 1 Representative functional (f)EIT video from patient 7 at time point T2 (mechanical ventilation at the start of surgery). ΔZ indicates the change in the impedance within the thorax during the respiratory cycle. It shows inhomogeneity of lung ventilation on the right–left plane. If reading the pdf online, click on the image to view the video. Video 1
