# Supplementary material for: Evaluation of lung homogeneity in neonates and small infants during general anaesthesia using electrical impedance tomography: a prospective observational study
Source: BJA Open. 2024 Sep 21;12:100344. doi: 10.1016/j.bjao.2024.100344 (PMC11447312; doi:10.1016/j.bjao.2024.100344)
Supplement: Multimedia component 1 [file mmc1.docx]

**Supplementary Materials
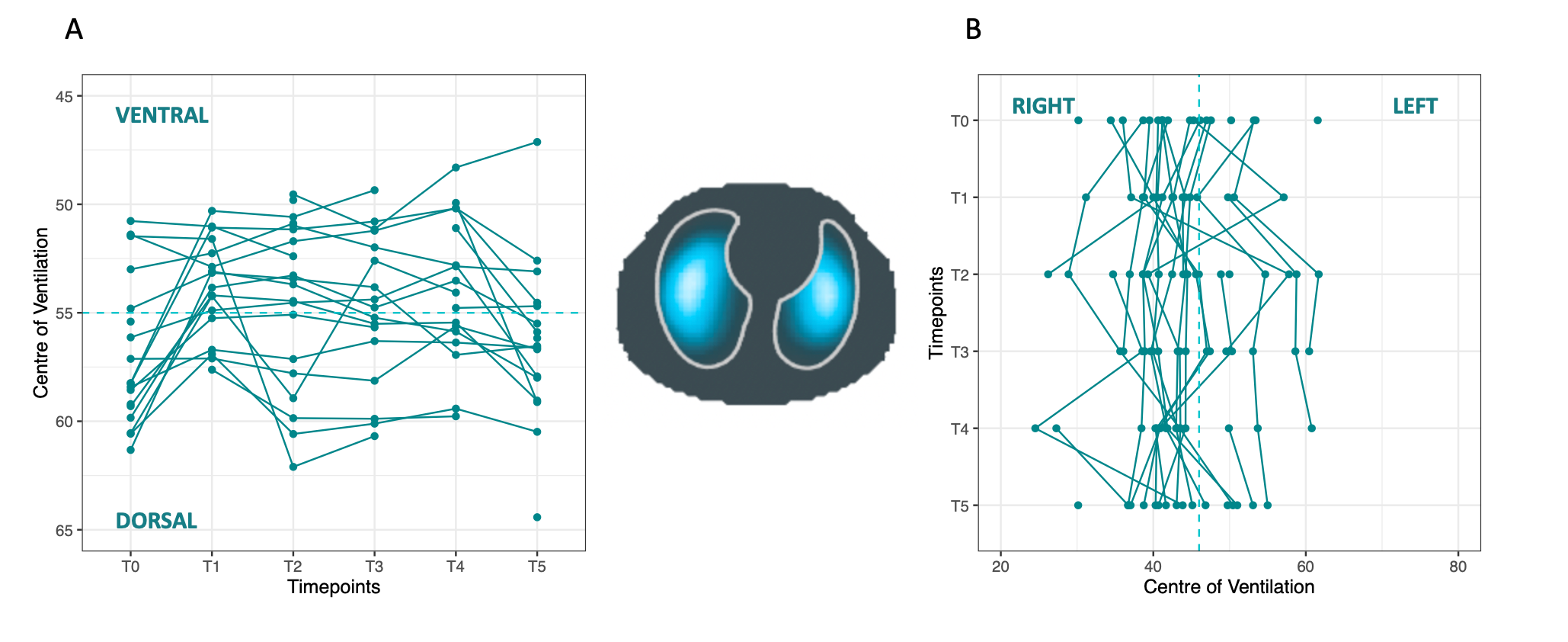
**

***Figure S1:***  Centre of ventilation (CoV) along the ventral-dorsal plane (A) and right-left plane (B) plotted for each patient at 6 anaesthesia timepoints (***T0*** spontaneous breathing with face mask at induction of anaesthesia; ***T1*** hand-bag ventilation via T-piece after intubation; ***T2*** mechanical ventilation at start of surgery; ***T3*** mechanical ventilation during surgery; ***T4*** mechanical ventilation at the end of surgery; ***T5*** spontaneous breathing after extubation). Dashed line represents the ideal CoV (55% for homogenous gravity-dependent ventilation, 46% for homogeneous right-left ventilation). These spaghetti plots suggest high inter-subject variability. It appears that ventilation distribution along the gravity dependent plane shifts ventrally from T0 to T1 to return more dorsally at T5 for most patients (A). Along right-left plane, ventilation distribution shows no clear trajectory at different anaesthesia timepoints.

Most CoV measurements are well below 46% (ideal CoV) suggesting a preferred ventilation in the right lung (B).

**A** **B**


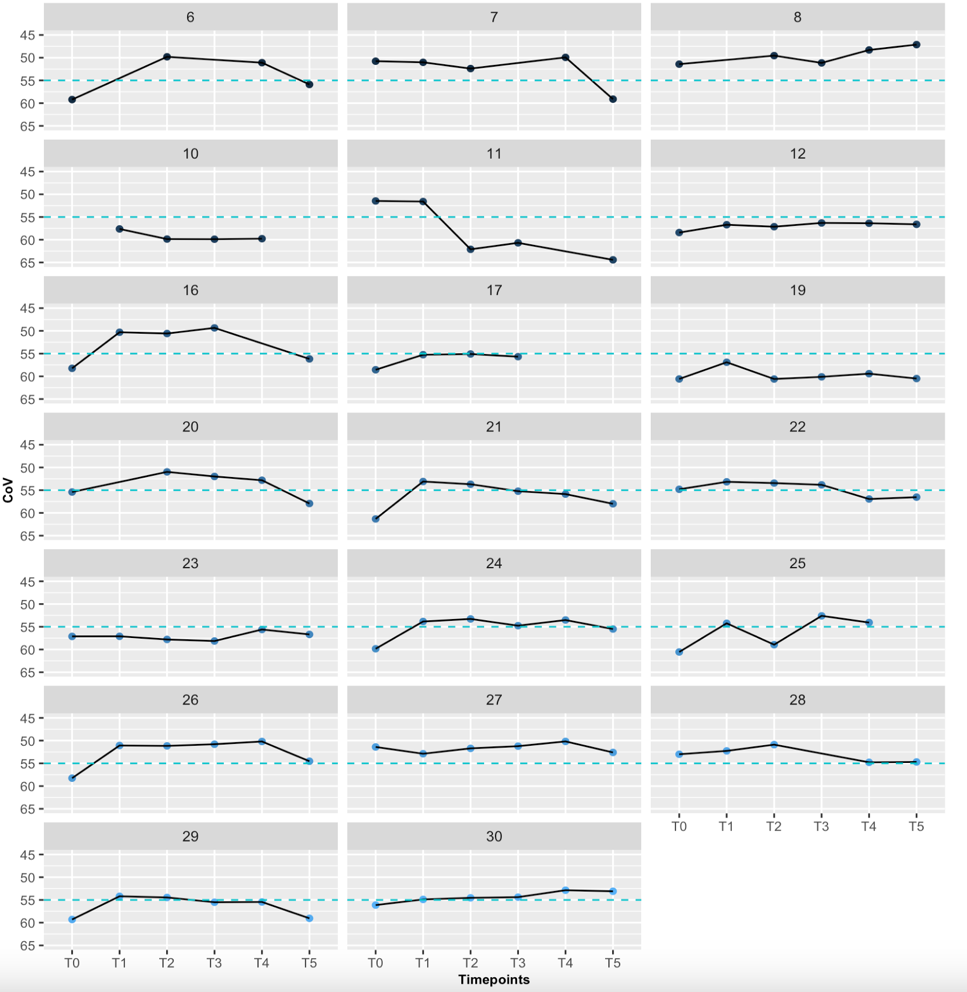

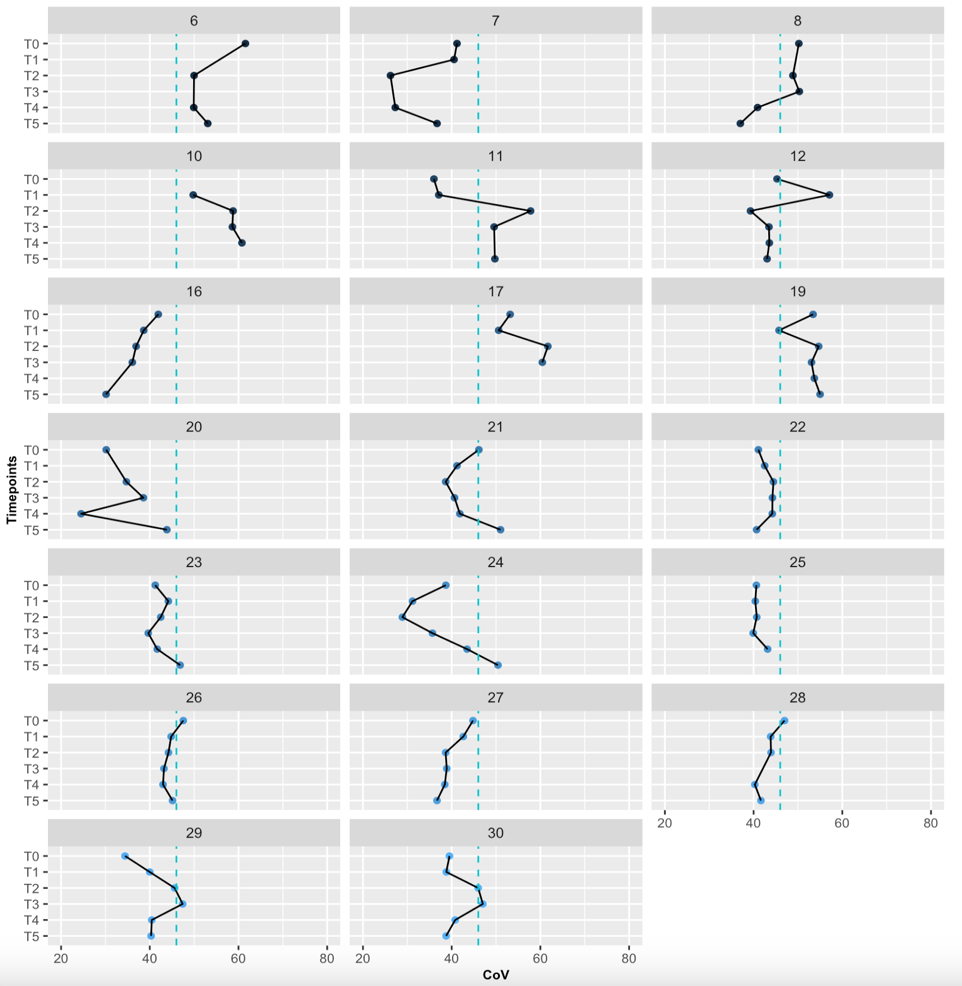


***Figure S2:***  Centre of ventilation (CoV) along the ventral-dorsal plane (CoV_VD_; A) and right-left plane (CoV_RL_; B) for every patient at six anaesthesia timepoints at 6 anaesthesia timepoints (***T0*** spontaneous breathing with face mask at induction of anaesthesia; ***T1*** hand-bag ventilation via T-piece after intubation; ***T2*** mechanical ventilation at start of surgery; ***T3*** mechanical ventilation during surgery; ***T4*** mechanical ventilation at the end of surgery; ***T5*** spontaneous breathing after extubation). Dashed line represents the ideal CoV (55% for homogenous gravity-dependent ventilation, 46% for homogeneous right-left ventilation). These single plots highlight the inter-subject variability and show the individual changes in the different timepoints.


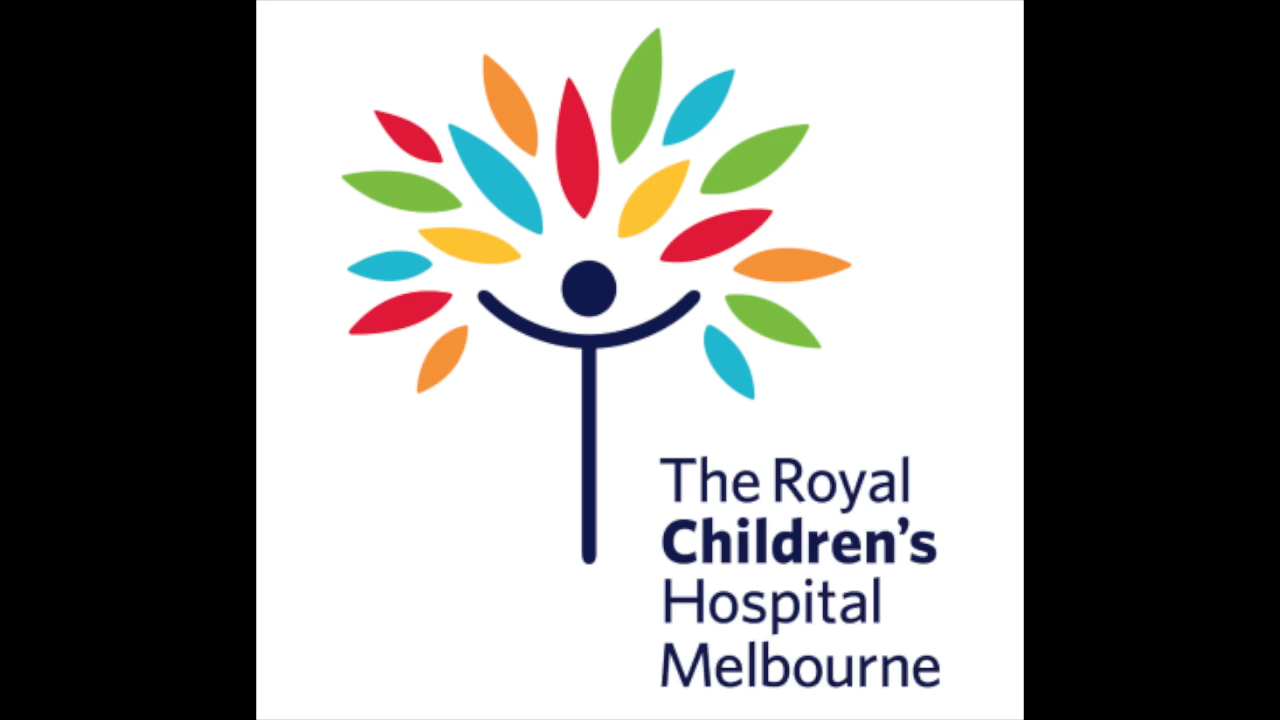


***Video S1***: Representative functional (f)EIT video from patient 7 at timepoint T2 (mechanical ventilation at start of surgery). ΔZ indicates the change in the impedance within the thorax during the respiratory cycle. It shows inhomogeneity of lung ventilation on right-left plane.
